# A long-term observational study on autoimmune pulmonary alveolar proteinosis revealed a sustained and generalized decrease in serum autoantibody levels

**DOI:** 10.1186/s13023-026-04274-w

**Published:** 2026-03-11

**Authors:** Etsuro Yamaguchi, Hiroyuki Tanaka, Eisuke Fujishiro, Masaya Fukami, Takuma Katano, Satoru Ito

**Affiliations:** Division of Respiratory Medicine and Allergology, Department of Internal Medicine, School of Medicine, Aichi Medical University, Yazako Karimata 1-1, Nagakute, Aichi Japan

**Keywords:** Autoimmune pulmonary alveolar proteinosis, Granulocyte-macrophage colony-stimulating factor, Autoantibody, Whole lung lavage, Disease severity score

## Abstract

**Backgrounds and objectives:**

Autoimmune pulmonary alveolar proteinosis (aPAP) is a rare lung disorder, and its long-term clinical outcomes, underlying determinants, and temporal dynamics of serum autoantibody levels remain poorly characterized.

**Methods:**

This single-center retrospective observational study comprised 64 patients diagnosed with aPAP, all of whom had been monitored for a minimum of three years following disease onset. Clinical courses were evaluated using disease severity score (DSS), based on arterial oxygen tension and respiratory symptoms. Serum anti–granulocyte-macrophage colony-stimulating factor (GM-CSF) IgG autoantibody (αGM) levels were quantified by enzyme-linked immunosorbent assay.

**Results:**

Among 64 patients with any DSS, 45.3% showed clinical improvement, 40.6% remained stable, and 14.1% experienced deterioration over median follow-up period of 7.8 years. In a binary logistic regression analysis excluding patients with an initial DSS of 1, for whom improvement was not feasible, higher initial DSS, higher baseline % forced vital capacity, and the absence of fibrotic patterns on computed tomography were identified as significant explanatory variables associated with DSS improvement, whereas whole lung lavage (WLL) and GM-CSF inhalation were not in the comparison of the initial and final DSS. Longitudinal assessment of serum αGM levels in 52 patients revealed a decline in 98% of them (*n* = 51) with an estimated antibody half-life of 3.5 years. GM-CSF inhalation therapy was significantly associated with lower final antibody levels adjusted for initial level and the interval between the initial and final measurements by analysis of covariance.

**Conclusions:**

The overall decline in αGM levels observed in this study may suggest a favorable or stable long-term prognosis in aPAP; however, further longitudinal investigations are warranted to confirm this presumed association.

**Supplementary Information:**

The online version contains supplementary material available at 10.1186/s13023-026-04274-w.

## Introduction

A quarter of a century has passed since the pivotal discovery of autoantibodies against granulocyte-macrophage colony-stimulating factor (GM-CSF) in patients with idiopathic pulmonary alveolar proteinosis (PAP) [1], leading to the reclassification of the disease as autoimmune pulmonary alveolar proteinosis (aPAP). Although substantial progress has been made in understanding the treatment [2–6] and genetic background [7] of aPAP, comprehensive data on its long-term clinical course remain limited. This scarcity is primarily attributable to the disease’s rarity, which hinders the accumulation of large patient cohorts. Additionally, the typically low mortality rate of aPAP—unlike other life-threatening pulmonary diseases—complicates the evaluation of long-term prognosis using conventional endpoints.

Previous studies have generally reported follow-up periods of approximately three years [8, 9], which may be insufficient given the often protracted and variable nature of aPAP. In this study, we present the long-term clinical course of 64 patients with aPAP who were followed for more than three years after disease onset at our institution. Clinical progression was assessed using the disease severity score (DSS), a composite index based on arterial oxygen tension and respiratory symptoms [8]. Furthermore, we longitudinally measured serum anti–GM-CSF IgG autoantibody (αGM) levels and observed a notable trend toward a gradual decline in antibody levels over time.

## Methods

### Study population

This study was a retrospective cross-sectional analysis of patients with aPAP conducted at Aichi Medical University Hospital, one of the tertiary referral centers for the diagnosis and therapy of aPAP in Japan. One hundred seven patients with aPAP were seen and followed up at our department from July 2003 to February 2024. One patient with aPAP and malignant lymphoma who underwent allogeneic bone marrow transplantation and chemotherapy was excluded because he had too complex pathophysiology to appropriately assess the clinical course. Excluding 42 patients who were followed up for less than 3 years since the onset, the remaining 64 patients were included in this study.

### Diagnosis

The diagnosis of PAP was made as previously described [8]. Briefly, they had characteristic and compatible findings on high-resolution computed tomography (HRCT) of the chest and either specific pathologic findings by lung biopsy or cytologic findings of bronchoalveolar lavage fluids or both. αGM levels had been measured at referring hospitals before the first visit to our hospital in a part of patients, and we ourselves measured them for the rest of patients as described below.

### Disease severity score (DSS)

Patients were assigned DSS based on the presence or absence of symptoms and the degree of reduction in PaO_2_ measured while patients were breathing room air as previously described [8]. The scoring system was as follows: DSS 1, asymptomatic with PaO₂ ≥ 70 Torr; DSS 2, symptomatic with PaO₂ ≥ 70 Torr; DSS 3, 60 ≤ PaO₂ < 70 Torr; DSS 4, 50 ≤ PaO₂ < 60 Torr; and DSS 5, PaO₂ < 50 Torr. In cases where PaO₂ was unavailable, peripheral oxygen saturation (SpO₂) measured by a pulse oximeter was used as a surrogate, with SpO₂ values of 93%, 90%, and 85% corresponding to PaO₂ values of 70, 60, and 50 Torr, respectively, based on a modified method from the previous study [8].

Clinical course was evaluated by comparing DSS at the first visit (initial DSS) to our hospital with that at the last visit (final DSS) during the follow-up periods. If DSS increased, remained unchanged, or decreased, the clinical course was judged as deteriorated, stable, or improved, respectively.

### Measurement of serum markers

Sera were collected at every outpatient visit as far as possible and stored at -80 °C. However, because it was not possible to collect sera regularly every year, we analyzed the results in the following time intervals; 52 patients at the first visit (initial measurement, T0), 49 after 3 to 5 years (3 ≦ T1 < 5), 37 after 5 to 10 years (5 ≦ T2 < 10), 14 after 10 to 15 years (10 ≦ T3 < 15), and 8 at the final measurement after T3 (T4).

αGM levels were measured by an in-house enzyme-linked immunosorbent assay which was designed to extensively reduce non-specific binding of IgG to assay plates in sera and to select optimal dilution ratios of sera to ensure that the absorbances of analyte are in the linear portion of the standard curve as previously described [10]. The 95th percentile value of healthy subjects and the discriminative value of aPAP from other diffuse lung diseases were 0.5 µg/ml and 2.8 µg/ml, respectively [10].

Serum levels of Krebs von den Lungen-6 (KL-6), the most dynamically changing serum marker of aPAP [11, 12], were measured by latex agglutination turbidimetric immunoassay using a commercially available kit (Nanopia KL-6 Reagent, SEKISUI MEDICAL CO., LTD., Tokyo, Japan).

### HRCT evaluation

Fibrotic patterns on HRCT were defined as the presence of traction bronchiectasis and/or honeycombing regardless of degree as previously reported [13].

### Statistical analysis

Continuous variables were presented as medians with interquartile range (IQR), and categorical variables as counts and percentages of the total, as appropriate. Normality was checked by the Shapiro-Wilk test. The Kruskal-Wallis test was used to compare follow-up periods across initial DSS groups. Fisher’s exact test was used to analyze the association between initial DSS and changes in final DSS, and distribution of DSS stratified by whether GM-CSF inhalation or WLL was performed or not.

Univariate logistic regression analysis was used for screening independent variables of improved clinical course defined by changes in DSS. Multivariate analysis was conducted by forward stepwise selection for variables with the p value less than 0.1 in univariate analysis. Multicollinearity was checked by variance inflation factor (VIF) calculated by linear regression analysis.

Comparisons of αGM levels across different time points were assessed by Wilcoxon signed-rank sum test. Analysis of covariance (ANCOVA) with the final αGM level as the dependent variable, categorical variables of patients’ characteristics as independent variables, and both the initial αGM level and the interval between initial and final measurements of antibody as covariates was performed to explore variables that potentially affect the decline of αGM levels.

All analyses were performed using IBM SPSS Statistics version 27 (Tokyo, Japan). A double-sided p value less than 0.05 was considered statistically significant.

## Results

### Demographics of study subjects

The study cohort comprised 64 patients with aPAP who were followed for a median of 7.8 years. Compared with a previous Japanese cohort [8], the present population showed a less pronounced male predominance (Table 1) [8]. A mild accumulation of autoimmune diseases other than aPAP was observed. Impairment of respiratory function at diagnosis was comparable to the earlier report [8].

**Table 1:** Demographic data at diagnosis and during the follow-up period

	Total number	*n* (%) or Median (IQR)
**At diagnosis**		
Age (year)	64	56.3 (47.1–64.1)
Gender (man/woman)	64	36 (56.3%)/28 (43.7%)
Smoking status (non/ex/current)	64	33 (51.6%)/23 (35.9%)/8 (12.5%)
Inhalation of occupational dust (yes/no)	64	1 (1.6%)/63 (98.4%)
Other comorbid autoimmune diseases (yes^a^/no)	64	8 (15.6%)/56 (84.4%)
%VC	63	86.9 (79.4–99.1)
%FVC	63	87.0 (76.7-100.3)
%FEV1	62	84.7 (75.8-103.4)
FEV1/FVC ratio (%)	62	81.9 (75.1–84.4)
%DLCO	55	74.3 (59.7–87.7)
Serum KL-6 levels (U/ml)	64	2833 (1661–5064)
Serum αGM levels (µg/ml)	64	26.6 (13.1–97.8)
**During the follow-up period**		
Follow-up periods from the onset (year)	64	7.8 (5.6–10.9)
GM-CSF inhalation (yes/no)	64	30 (46.9%)/34 (53.1%)
Inhalation period (month)	64	12.0 (6.0–18.0)
Use of corticosteroids (yes^b^/no)	64	6 (9.4%)/58 (90.6%)
Whole lung lavage (yes/no)	64	29 (45.3%)/35 (54.7%)
Number of lavaged lung	64	2.0 (2.0–4.0)
Years from the onset to the last lavage	64	3.6 (1.1–5.5)
Fibrotic patterns on HRCT (yes/no)	64	18 (28.1%)/46 (71.9%)
Long-term oxygen therapy (yes/no)	64	20 (31.3%)/44 (68.7%)

Abbreviations: IQR, interquartile range; %VC, percentage of predicted vital capacity; %FVC, percentage of predicted forced vital capacity; %FEV1, percentage of predicted forced expiratory volume in one second; %DLCO, percentage of predicted diffusing capacity of the lung for carbon monoxide; KL-6, Krebs von den Lungen-6; αGM, anti-GM-CSF IgG antibody; HRCT, high resolution computed tomography. ^a^ 3 with rheumatoid arthritis, 1 with polymyalgia rheumatica, 1 with dermatomyositis, 1 with polymyositis, 1 with Behçet’s disease, 1 with autoimmune hepatitis. ^b^ 2 with rheumatoid arthritis, 1 with polymyalgia rheumatica, 1 with dermatomyositis, 1 with polymyositis, 1 with autoimmune hepatitis

### Clinical courses

During the median follow-up, 46.9% of patients received the inhalation therapy with GM-CSF (Table 1). Most participated in clinical trials involving sargramostim or molgramostim [4, 5, 14], while others received molgramostim off-label. Some patients were administered systemic corticosteroids for comorbid autoimmune diseases. A significant proportion of patients (45.3%) were subject to whole lung lavage (WLL), reflecting that our hospital is a tertiary referral institution. Most patients underwent bilateral WLL on two separate days. Most WLL were conducted within approximately five years of onset (median 3.6 years; IQR, 2.1–5.5 years). Fibrotic patterns on HRCT developed in 28.1% of patients during the follow-up periods. Comparable proportion of patients underwent long-term oxygen therapy. Uncontrolled pulmonary infections were not observed. Death due to respiratory failure caused by pulmonary fibrosis occurred in three patients (4.7%) 3.3, 7.0, and 9.2 years after the onset of aPAP. One patient received plasmapheresis and rituximab in addition to GM-CSF inhalation and WLL. The other one patient had concurrent dermatomyositis. Nintedanib was administered to one patient; however, no clinical benefit was observed.

### Longitudinal changes in DSS

Clinical courses assessed by final DSS according to initial DSS are presented in Table 2. There was no significant difference in follow-up periods among five initial DSS groups. If clinical remission is defined as DSS 1 irrespective of CT findings and/or lung function, 28 (43.8%) of all patients maintained or improved to remission during the follow-up periods (Table 2). Spontaneous remission, defined as the achievement or maintenance of DSS 1 without major treatments, such as WLL and/or GM-CSF inhalation, was observed in 13 of 23 (56.5%) (Table S1, supplementary material) untreated patients. Changes in DSS were significantly associated with initial DSS (Table 2); patients with high initial DSS were more likely to improve. This result can be explained simply by the fact that patients with initial DSS of 1 could not improve any further, and those with higher initial DSS naturally received more extensive treatments, such as WLL and GM-CSF inhalation, leading to improvement. Therefore, further analysis was limited to the subpopulation with initial DSS 2 or more (the shaded area in Table 2).

**Table 2:**
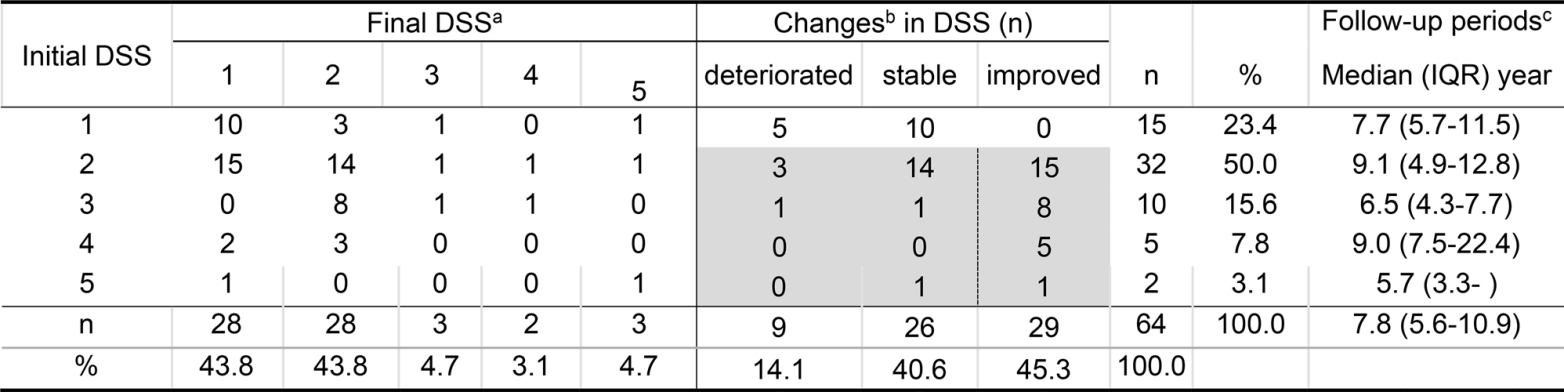
Final DSS according to initial DSS and the length of follow-up periods

Abbreviations: DSS, disease severity score; IQR, interquartile range

^a^ *p* = 0.015 by Fisher’s exact test

^c^ *p* = 0.14 by Kruskal-Wallis test

DSS: disease severity score; ^b^ deteriorated, stable, or improved changes in DSS means that DSS increased, remained unchanged, or decreased, respectively. The shaded area was the subject of binary logistic regression analysis and the dashed line indicates the boundary between two groups, i.e., improved vs. not improved

### Determinants for clinical courses

To conduct binary logistic regression for clinical courses, the dependent variable was defined as either DSS was improved or not (deteriorated or stable) (Table 2). There was a high degree of collinearity for percentage of predicted vital capacity (%VC) (VIF, 21.7) and percentage of predicted forced vital capacity (%FVC) (VIF, 25.8), and modest collinearity for percentage of predicted forced expiratory volume in one second (%FEV1) (VIF, 6.5) in Table 1; therefore, %VC and %FEV1 were excluded from independent variables. In the univariate logistic regression with the other variables as independent variables, three variables showed p values less than 0.1 (Table 3). VIF for fibrotic patterns on CT, %FVC, and initial DSS were 1.003, 1.004, and 1.002, respectively, indicating minimal collinearity. These variables were included in the multivariate logistic regression analysis, and all were found to be significant explanatory variables (Table 3).

**Table 3:** Binary logistic regression analysis of improved DSS for patients with initial DSS of 2 or more

	Univariate				Multivariate		
	reference	odds ratio	95% CI	*p* value	odds ratio	95% CI	*p* value
Gender	man	0.622	0.196–1.972	0.420			
Age at onset		0.991	0.955–1.027	0.609			
Smoking status	non-smoker	0.803	0.343–1.880	0.613			
Initial DSS	DSS 2	2.342	0.939–5.839	0.068	5.538	1.406–21.813	0.014
Comorbid autoimmune disease	none	0.462	0.091–2.335	0.350			
Fibrotic patterns on HRCT	none	0.208	0.057–0.766	0.018	0.036	0.003–0.487	0.012
%FVC at diagnosis		1.073	1.020–1.127	0.006	1.098	1.029–1.171	0.004
FEV1/FVC at diagnosis		0.995	0.921–1.075	0.905			
%DLCO at diagnosis		1.014	0.978–1.051	0.442			
Serum KL-6 levels at diagnosis	100 U/ml increase	0.999	0.993–1.005	0.702			
Serum KL-6 levels at final visit	100 U/ml increase	0.984	0.962–1.007	0.176			
Serum αGM levels at diagnosis		0.998	0.994–1.002	0.347			
Serum αGM levels at final visit		0.993	0.978–1.009	0.399			
GM-CSF inhalation	not done	1.071	0.343–3.349	0.906			
Corticosteroids therapy	not done	3.375	0.554–20.551	0.187			
Whole lung lavage	not done	0.764	0.243–2.395	0.764			
Long-term oxygen therapy	not done	0.789	0.243–2.563	0.694			
Follow-up periods from the onset		0.994	0.882–1.190	0.915			

A﻿bbreviations: DSS, disease severity score; CI, confidence interval; HRCT, high-resolution computed tomography; %FVC, percentage of predicted forced vital capacity; FEV1, forced expiratory volume in one second; %DLCO, percentage of predicted diffusing capacity of the lung for carbon monoxide; KL-6, Krebs von Den Lungen-6; αGM, anti-GM-CSF IgG autoantibody

Serum levels of KL-6 and αGM at both diagnosis and final visit were not associated with the clinical outcome (Table 3). Similar results were obtained for patients with all initial DSS (Table S2, supplementary material). Meanwhile, similar analysis for all patients revealed that final serum KL-6 levels were associated with mortality due to aPAP (Table S3, supplementary material).

In the primary analysis as described above, GM-CSF inhalation therapy and WLL did not appear to influence the clinical course; however, we conducted a more in-depth investigation. In general, DSS fluctuates over time, and both treatments were typically initiated in patients whose condition was worsening. Indeed, when we examined the distribution of DSS at the initial visit, at the time of worst severity, and at the final visit according to the presence or absence of each treatment, we found that the maximum DSS in the treatment groups was skewed toward more severe categories compared with the groups that did not receive these treatments (Table S4, supplementary material). Based on these findings, we conducted a secondary analysis to evaluate the effect of inhalation therapy or WLL on the improvement in DSS between the period of worst severity and the final visit. Binary logistic regression analysis with improvement status (improved vs. not improved) as the dependent variable revealed that inhalation therapy was a significant variable (odds ratio 5.750, 95% CI 1.076–30.720, *p* = 0.041), whereas WLL did not (odds ratio 3.020, 95% CI 0.678–13.442, *p* = 0.147).

### Longitudinal changes in antibody levels

**Fig. 1:**
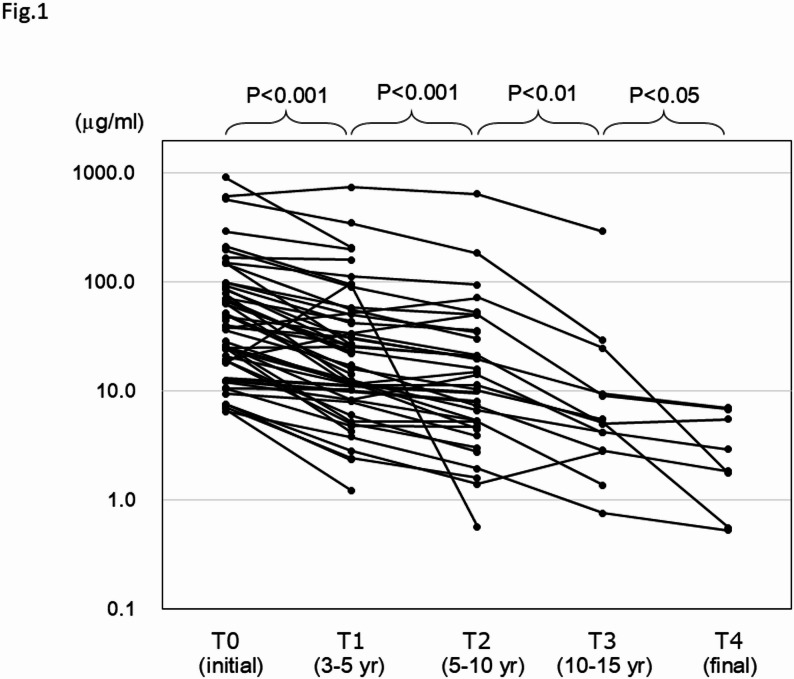
Anti-granulocyte-macrophage colony-stimulating factor IgG autoantibody levels in patients with autoimmune pulmonary alveolar proteinosis T0, at the first visit (initial measurement); T1, 3 to 5 years after T0; T2, 5 to 10 years after T0; T3, 10 to 15 years after T0; T4, final measurement after T3

**Fig. 2:**
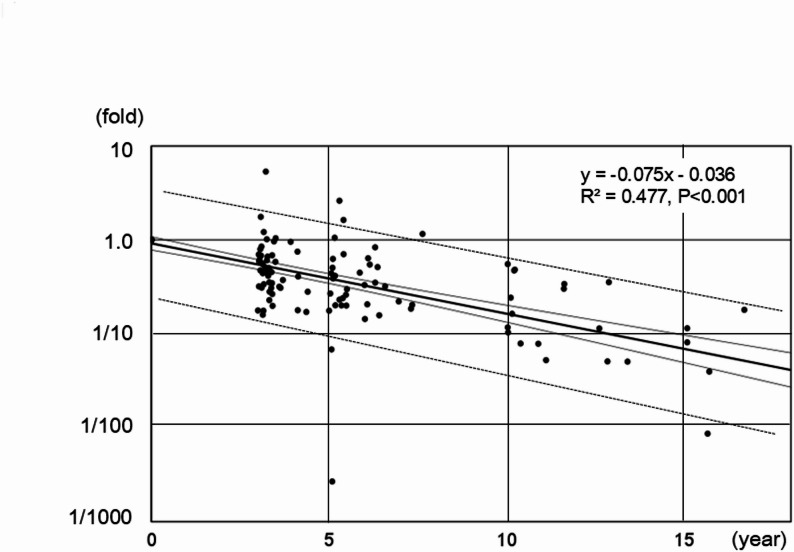
Linear regression of anti-granulocyte-macrophage colony-stimulating factor (GM-CSF) IgG autoantibody levels. The ratios of anti-GM-CSF IgG autoantibody levels at each assay time point relative to the initial levels are plotted on a logarithmic scale, expressed in fold change. Note that all fold ratios at 0 year are equal to 1.0 and overlapping. Thick line, regression line; thin solid lines, the 95% confidence interval of the mean; doted lines, the 95% prediction interval of a single future antibody level

Sera were collected in a significant proportion of patients from the first visit to our hospital and thereafter according to the follow-up periods. Serum αGM levels were measured and pairwise comparisons of αGM levels between adjacent time intervals revealed consistent and significant decrease (Fig. 1). Transiently elevated but finally decreased levels compared with the baseline levels were observed for seven patients. One patient exceptionally showed a final increase in the second assay, conducted 3.1 years after the initial assay. All the other patients showed consistent decrease in αGM levels. Ultimately, a final decline in autoantibody levels was observed in 51 of 52 (98%) patients during the follow-up periods. The overall apparent decline was modeled using linear regression, with the logarithmic ratio of αGM levels to their baseline values plotted on the vertical axis and the corresponding measurement years on the horizontal axis. (Fig. 2). Based on the regression equation, the half-life of αGM level was estimated to be 3.5 years. The durations required for the initial αGM levels to decline to the reported upper and lower autoantibody thresholds of active aPAP [15] were calculated to be 2.1 and 4.4 years, respectively.

To investigate the impact of patients’ baseline characteristics, longitudinal clinical features, and treatment modalities on the reduction of antibody levels, we used ANCOVA with the final αGM level as the dependent variable adjusted for both the initial αGM level and the interval between the initial and the final measurements, and each categorical variable in Table 1 as the independent variable. Among the variables examined, GM-CSF inhalation therapy was the only factor found to be significantly associated with lower final antibody levels (Table 4). The adjusted mean αGM level was 19.2 (95% CI, 4.5–33.8) µg/ml in patients who received GM-CSF inhalation therapy and 45.1 (95% CI, 30.1–60.1) µg/ml in those who did not.

**Table 4:** Analysis of covariance examining factors influencing the final αGM level, with initial αGM level and interval between the first and final measurements as covariates

Independent variables	Sums of squares	Degree of freedom	Mean square	F value	*p* value
Gender	4290.1	1	4290.1	2.96	0.09
Smoking status	1450.2	2	725.1	0.47	0.62
Comorbid autoimmune diseases	1484.8	1	1484.8	0.98	0.33
GM-CSF inhalation	8307.6	1	8307.6	6.09	0.02
Use of corticosteroids	1014.8	1	1014.8	0.67	0.41
Whole lung lavage	668.8	1	668.8	0.44	0.51
Fibrotic patterns on HRCT	5155.8	1	5155.8	3.60	0.06
Long-term oxygen therapy	46.3	1	46.3	0.03	0.86
Initial DSS	6464.7	4	1616.2	1.08	0.38
Final DSS	1084.7	3	542.3	0.35	0.71
Changes in DSS (improved or not)	2683.9	1	2683.9	1.81	0.19

Abbreviations: αGM, anti-granulocyte-macrophage colony-stimulating factor IgG autoantibody; HRCT, high resolution computed tomography; DSS, disease severity score

## Discussion

We conducted an observational study on aPAP in a significant number of Japanese patients for a median observational time of 7.8 years. Despite retrospective and single-center observation, this study included all the patients followed-up for more than 3 years except for only one patient with dual pathogenetic components of autoimmune and secondary to malignant lymphoma, and had the advantage of including detailed experience accumulated over several years. In this report, we have focused on two aims: firstly, the description of the clinical courses assessed by DSS and the factors responsible for clinical outcomes; secondly, to clarify changes in αGM levels for a long period of time. The latter has not been covered in the literature so far.

There have been several reports on the prognosis of pulmonary alveolar proteinosis; however, most reports covered the period before the era of autoantibodies [16, 17]. Only recent short-term reports on patients diagnosed with aPAP are worth comparing with ours [8, 9, 18, 19]. The rate of improved cases in the present study (45.3%) seemed better than that previously reported for Japanese cases (30%) with median follow-up of 10 months [8], but comparable to that in a Germany cohort (40%) with a mean follow-up of 3.1 years [9]. The factors responsible for favorable clinical courses in our study included good %FVC at diagnosis and high initial DSS. These findings suggest that patients with preserved lung function are more likely to improve, and that those with severely affected individuals are more likely to improve with intensive treatment. As anticipated, the development of fibrotic patterns on HRCT during the follow-up periods was unfavorable clinical outcome, consistent with a recent report [19].

Contrary to expectations, GM-CSF inhalation and WLL were not significant variables for better outcome evaluated across the first and last visits. However, inhalation therapy was associated with improved DSS between the period of worst clinical status and the final visit. These findings suggest that although the therapy may contribute to short-term improvement, it may not influence long-term outcomes. A prolonged treatment regimen with a higher dose would be highly expected to improve long-term outcomes.

Across both comparison periods (initial vs. final, worst vs. final) and when evaluated in terms of mortality, WLL was not associated with improved prognosis in our cohort. These results appear to be inconsistent with the prior large meta-analysis, which demonstrated that the patients with acquired PAP who underwent WLL had a superior survival [17]. Two possible reasons can be considered. First, therapeutic lavage is not targeted to the root pathophysiology, the production of autoantibodies. Second, the elevated DSS that prompted WLL may have diminished their therapeutic efficacy. Since the short-term efficacy of WLL has been well established, and in some cases, it confers a life-saving benefit, the present findings do not undermine the clinical significance of WLL.

Meanwhile, a clear overall decline in αGM levels was observed for nearly all patients. Decreased serum levels of autoantibody at remission compared with those at diagnosis have been reported for a part of patient population [9]. We have for the first time found a remarkable downward trend in serum αGM levels for significant number of aPAP patients over an extended follow-up period than ever before.

Robust evidence has verified a causal link between autoantibodies and the pathogenesis of aPAP. Firstly, a markedly elevated levels of autoantibodies are detected in the serum obtained from patients with aPAP, exceeding the range observed in healthy individuals [1, 8, 10]. Secondly, Patient-derived autoantibodies reproduced pulmonary alveolar proteinosis in nonhuman primates [20]. Thirdly, suppression of autoantibody production by rituximab or inhaled GM-CSF that counteracts autoantibodies have shown clinical efficacy [4, 6, 21]. Given this evidence relevant to causality, a strong correlation between antibody levels and disease severity would be expected; however prior studies [8, 9] and our own data (Table 3, Table S2) do not support this assumption.

The critical threshold of serum GM-CSF autoantibodies associated with active aPAP has been proposed [15, 22]. Remission or inactive state of aPAP, as the inverse of disease onset, is theoretically anticipated when serum autoantibody levels decline below the critical threshold. However, this threshold is likely to exhibit substantial interindividual variability, influenced by factors such as antibody affinity, local concentrations of GM-CSF in alveolar milieu, and the responsiveness of alveolar macrophages to GM-CSF. These biological variabilities may underlie the limited reliability of serum autoantibody levels as predictive biomarkers for disease activity or remission timing. Nevertheless, the following findings may offer practical insights. The median year from the onset to the last WLL required for treatment in this study was 3.6 years. The estimated half-life of αGM levels, based on the model in Fig. 2 was 3.5 years. The time required for αGM levels to decline to the presumed threshold [15] was estimated to be 2.1 to 4.4 years from the initial assay in our cases. The mean length of time to remission has been reported to be 3.7 years, although the definition of remission was not the same as ours [9].

Taken together, these findings suggest that in most surviving patients, aPAP eventually enters a quiescent phase—either spontaneously or following a variable period of treatment with WLL and/or GM-CSF inhalation. Timely and appropriate use of these therapies may be critical in preventing fibrotic progression, which is associated with poor outcomes. However, a subset of patients may experience slow disease progression, and further research is needed to understand the trajectory of antibody levels in these individuals.

Another intriguing finding in this study was the greater reduction of final antibody levels observed for patients who received inhaled GM-CSF therapy. This phenomenon may reflect a desensitization effect as reported in patients with Fabry disease treated with enzyme replacement therapy [23]. Nonetheless, due to limited sample size and heterogeneity in treatment regimens, this result should be interpreted with caution and validated through more stringent analyses. At the very least, the fact that this therapy does not increase antibody levels is of considerable importance.

An additional noteworthy observation is that the declining trend in antibody levels appeared remarkably robust, showing no apparent influence from patient backgrounds. This phenomenon may be related to fundamental mechanisms underlying antibody production. In this context, it has been known that several infections induce autoantibodies, some of which are associated with clinical disease manifestations [24, 25]. Notably, recent detailed investigations on coronavirus disease 2019 (COVID-19) have demonstrated the induction of functional autoantibodies against a broad range of extracellular or secreted proteins, including GM-CSF [26]. These autoantibodies persist in the circulation for more than one year, although their overall levels tend to decline over time [27]. The onset of disease in our study cohort occurred prior to the COVID-19 pandemic; however, respiratory infections caused by non–SARS-CoV-2 (severe acute respiratory syndrome coronavirus 2) coronaviruses or other viruses are common [28]. It is conceivable that such infections, in combination with the predisposition we identified [7] and endogenous human herpesvirus 6 [29], may have contributed to the induction of autoantibodies against GM-CSF.

This study has certain limitations, including its retrospective nature, single-center setting, and limited sample size. Nonetheless, these conditions enabled the acquisition of precise clinical data. In addition, clinical progression was evaluated solely using DSS, rather than pulmonary function parameters or radiological findings. DSS, a composite index of symptoms and PaO₂, has been validated in prior studies [8, 12] and correlated well with pulmonary diffusing capacity [8], a primary endpoint adopted in a recent clinical trial [6]. Given that each evaluative measure has inherent strengths and weaknesses, we highlight the importance of incorporating diverse perspectives, such as quality-of-life questionnaire scores, to better capture the complexity of disease trajectories. However, achieving consensus among researchers could be challenging.

In conclusion, this study demonstrated a general decline in serum autoantibody levels over a long-term follow-up period, which is clinically intriguing. More importantly, it highlights the critical need to elucidate the underlying immunological mechanisms why anti-GM-CSF autoantibodies are initially produced yet subsequently decline spontaneously.

## Supplementary Information

Below is the link to the electronic supplementary material.

**Supplementary Material 1**:Title: Proportion of final DSS 1 according to initial DSS in patients without treatment with WLL and/or GM-CSF inhalation during the follow-up periods. Description: As described in the text.

**Supplementary Material 2**: Title: Binary logistic regression analysis of improved DSS for all patients Description: As described in the text.

**Supplementary Material 3**: Title: Binary logistic regression analysis of death due to aPAP for all patients Description: As described in the text.

**Supplementary Material 4**: Title: Distribution of DSS stratified by each treatment modality and assessment phase in patients with initial DSS>1. Description: As described in the text.

## Data Availability

The datasets used and/or analyzed during the current study are available from the corresponding author on reasonable request.

## Abbreviations

GM-CSF: Granulocyte-macrophage colony-stimulating factor
PAP: Pulmonary alveolar proteinosis
aPAP: Autoimmune pulmonary alveolar proteinosis
DSS: Disease severity score
αGM: Anti-GM-CSF IgG autoantibody
HRCT: High-resolution computed tomography
KL-6: Krebs von den Lungen-6
IQR: Interquartile range
WLL: Whole lung lavage
%VC: Percentage of vital capacity
VIF: Variance inflation factor
%FVC: Percentage of forced vital capacity
%FEV1: Percentage of forced expiratory volume in one second

## References

[1] Kitamura T, Tanaka N, Watanabe J, Uchida K, Kanegasaki S, Yamada Y, et al. Idiopathic pulmonary alveolar proteinosis as an autoimmune disease with neutralizing antibody against granulocyte/macrophage colony-stimulating factor. J Exp Med. 1999;190(6):875–80. 10.1084/jem.190.6.875. 10499925 10.1084/jem.190.6.875 PMC2195627

[2] McCarthy C, Bonella F, O’Callaghan M, Dupin C, Alfaro T, Fally M, et al. European respiratory society guidelines for the diagnosis and management of pulmonary alveolar proteinosis. Eur Respir J. 2024;64(5):2400725. 10.1183/13993003.00725-2024. 39147411 10.1183/13993003.00725-2024

[3] Trapnell BC, Nakata K, Bonella F, Campo I, Griese M, Hamilton J, et al. Pulmonary alveolar proteinosis. Nat Rev Dis Primers. 2019;5(1):16. 10.1038/s41572-019-0066-3. 30846703 10.1038/s41572-019-0066-3

[4] Tazawa R, Ueda T, Abe M, Tatsumi K, Eda R, Kondoh S, et al. Inhaled GM-CSF for pulmonary alveolar proteinosis. N Engl J Med. 2019;381(10):923–32. 10.1056/NEJMoa1816216. 31483963 10.1056/NEJMoa1816216

[5] Trapnell BC, Inoue Y, Bonella F, Morgan C, Jouneau S, Bendstrup E, et al. Inhaled Molgramostim therapy in autoimmune pulmonary alveolar proteinosis. N Engl J Med. 2020;383(17):1635–44. 10.1056/NEJMoa1913590. 32897035 10.1056/NEJMoa1913590 PMC8083051

[6] Trapnell BC, Inoue Y, Bonella F, Wang T, McCarthy C, Arai T, et al. Phase 3 trial of inhaled Molgramostim in autoimmune pulmonary alveolar proteinosis. N Engl J Med. 2025;393(8):764–73. 10.1056/NEJMoa2410542. 40834301 10.1056/NEJMoa2410542

[7] Sakaue S, Yamaguchi E, Inoue Y, Takahashi M, Hirata J, Suzuki K, et al. Genetic determinants of risk in autoimmune pulmonary alveolar proteinosis. Nat Commun. 2021;12(1):1032. 10.1038/s41467-021-21011-y. 33589587 10.1038/s41467-021-21011-y PMC7884840

[8] Inoue Y, Nakata K, Arai T, et al. Characteristics of a large cohort of patients with autoimmune pulmonary alveolar proteinosis in Japan. Am J Respir Crit Care Med. 2005;177(7):752–62. 10.1164/rccm.200708-1271OC. 10.1164/rccm.200708-1271OC PMC2720118 18202348

[9] Bonella F, Bauer PC, Griese M, Ohshimo S, Guzman J, Costabel U. Pulmonary alveolar proteinosis: new insights from a single-center cohort of 70 patients. Respir Med. 2011;105(12):1908–16. 10.1016/j.rmed.2011.08.018. 21900000 10.1016/j.rmed.2011.08.018

[10] Nishimura M, Yamaguchi E, Takahashi A, Asai N, Katsuda E, Ohta T, et al. Clinical significance of serum anti-GM-CSF autoantibody levels in autoimmune pulmonary alveolar proteinosis. Biomark Med. 2018;12(2):151–9. 10.2217/bmm-2017-0362. 29202602 10.2217/bmm-2017-0362

[11] Takahashi T, Munakata M, Suzuki I, Kawakami Y. Serum and Bronchoalveolar fluid KL-6 levels in patients with pulmonary alveolar proteinosis. Am J Respir Crit Care Med. 1998;158(4):1294–8. 10.1164/ajrccm.158.4.9712003. 9769294 10.1164/ajrccm.158.4.9712003

[12] Bonella F, Ohshimo S, Miaotian C, Griese M, Guzman J, Costabel U. Serum KL-6 is a predictor of outcome in pulmonary alveolar proteinosis. Orphanet J Rare Dis. 2013;8:53. 10.1186/1750-1172-8-53. 23557396 10.1186/1750-1172-8-53 PMC3629718

[13] Akira M, Inoue Y, Arai T, Sugimoto C, Tokura S, Nakata K, et al. Pulmonary fibrosis on high-resolution CT of patients with pulmonary alveolar proteinosis. Am J Roentgenol. 2016;207(3):544–51. 10.2214/ajr.15.14982. 27548000 10.2214/AJR.15.14982

[14] Tazawa R, Trapnell BC, Inoue Y, Arai T, Takada T, Nasuhara Y, et al. Inhaled granulocyte/macrophage-colony stimulating factor as therapy for pulmonary alveolar proteinosis. Am J Respir Crit Care Med. 2010;181(12):1345–54. 10.1164/rccm.200906-0978oc. 20167854 10.1164/rccm.200906-0978OC PMC2894410

[15] Uchida K, Nakata K, Suzuki T, Luisetti M, Watanabe M, Kochet DE, et al. Granulocyte/macrophage-colony-stimulating factor autoantibodies and myeloid cell immune functions in healthy subjects. Blood. 2009;113(11):2547–56. 10.1182/blood-2009-05-155689. 19282464 10.1182/blood-2009-05-155689 PMC2656275

[16] Rosen SH, Castleman B, Liebow AA, Enzinger FM, Hunt RTN. Pulmonary alveolar proteinosis. N Engl J Med. 1958;258(23):1123–42. 10.1056/NEJM195806052582301. 13552931 10.1056/NEJM195806052582301

[17] Seymour JF, Presneill JJ. Pulmonary alveolar proteinosis: progress in the first 44 years. Am J Respir Crit Care Med. 2002;166(2):215–35. 10.1164/rccm.2109105. 12119235 10.1164/rccm.2109105

[18] Fijołek J, Wiatr E, Radzikowska E, Bestry I, Langfort R, Polubiec-Kownacka M, et al. Pulmonary alveolar proteinosis during a 30-year observation. Diagnosis and treatment. Pneumonol Allergol Pol. 2014;82(3):206–17. 10.5603/PiAP.2014.0028. 10.5603/PiAP.2014.0028 24793148

[19] Guirriec Y, Luque-Paz D, Bernard G, Mabo A, Kerjouan M, Ménard C, et al. Pulmonary fibrosis in patients with autoimmune pulmonary alveolar proteinosis: a retrospective nationwide cohort study. ERJ Open Res. 2024;10(6):00314–2024. 10.5603/PiAP.2014.0028. 39624377 10.1183/23120541.00314-2024 PMC11610044

[20] Sakagami T, Beck D, Uchida K, Uchida K, Suzuki T, Carey BC, Nakata K, et al. Patient-derived granulocyte/macrophage colony-stimulating factor autoantibodies reproduce pulmonary alveolar proteinosis in nonhuman primates. Am J Respir Crit Care Med. 2010;182(1):49–61. 10.1164/rccm.201001-0008OC. 20224064 10.1164/rccm.201001-0008OC PMC2902758

[21] Kavuru MS, Malur A, Marshall I, Barna BP, Meziane M, Huizar I, et al. An open-label trial of rituximab therapy in pulmonary alveolar proteinosis. Eur Respir J. 2011;38(6):1361–7. 10.1183/09031936.00197710. 21478218 10.1183/09031936.00197710 PMC3874725

[22] Uchida K, Nakata K, Carey B, Chalk C, Suzuki T, Sakagami T, et al. Standardized serum GM-CSF autoantibody testing for the routine clinical diagnosis of autoimmune pulmonary alveolar proteinosis. J Immunol Methods. 2014;402(1–2):57–70. 10.1016/j.jim.2013.11.011. 24275678 10.1016/j.jim.2013.11.011 PMC7985885

[23] Kubota T, Tsukimura T, Shiga T, Togawa T, Sakuraba H. Monitoring of anti-drug antibodies and disease-specific biomarkers in three patients from a Japanese Fabry family treated with enzyme replacement therapy. CEN Case Rep. 2023;12(2):171–5. 10.1007/s13730-022-00738-7. 36205882 10.1007/s13730-022-00738-7 PMC10151431

[24] Soldan SS, Lieberman PM. Epstein-Barr virus and multiple sclerosis. Nat Rev Microbiol. 2022;21(1):51–64. 10.1038/s41579-022-00770-5. 35931816 10.1038/s41579-022-00770-5 PMC9362539

[25] Leonhard SE, Papri N, Querol L, Rinaldi S, Shahrizaila N, Jacobs BC. Guillain-Barre syndrome. Nat Rev Dis Primers. 2024;10(1):97. 10.1038/s41572-024-00580-4. 39702645 10.1038/s41572-024-00580-4

[26] Wang EY, Mao T, Klein J, Dai Y, Huck JD, Jaycox JR, et al. Diverse functional autoantibodies in patients with COVID-19. Nature. 2021;595(7866):283–8. 10.1038/s41586-021-03631-y. 34010947 10.1038/s41586-021-03631-y PMC13130511

[27] Jernbom AF, Skoglund L, Pin E, Sjöberg R, Tegel H, Hober S, et al. Prevalent and persistent new-onset autoantibodies in mild to severe COVID-19. Nat Commun. 2024;15(1):8941. 10.1038/s41467-024-53356-5. 39414823 10.1038/s41467-024-53356-5 PMC11484904

[28] Iyadorai T, Lim SH, Wong PL, Sii HL, P’ng CK, Ee SS, et al. Clinical symptoms, comorbidities and health outcomes among outpatients infected with the common cold coronaviruses versus influenza virus. Virol J. 2024;21(1):251. 10.1186/s12985-024-02524-6. 39380036 10.1186/s12985-024-02524-6 PMC11462790

[29] Sasa N, Kojima S, Koide R, Hasegawa T, Namkoong H, Hirota T, et al. Blood DNA Virome associates with autoimmune diseases and COVID-19. Nat Genet. 2025;57(1):65–79. 10.1038/s41588-024-02022-z. 39753770 10.1038/s41588-024-02022-z PMC11735405

