# Supplementary material for: A long-term observational study on autoimmune pulmonary alveolar proteinosis revealed a sustained and generalized decrease in serum autoantibody levels
Source: Orphanet J Rare Dis. 2026 Mar 11;21:190. doi: 10.1186/s13023-026-04274-w (PMC13159374; doi:10.1186/s13023-026-04274-w)
Supplement: Supplementary file 1 — Supplementary Material 1:Title: Proportion of final DSS 1 according to initial DSS in patients without treatment with WLL and/or GM-CSF inhalation during the follow-up periods. Description: As described in the text. [file 13023_2026_4274_MOESM1_ESM.docx]

**Table S1** Proportion of final DSS 1 according to initial DSS in patients without treatment with whole lung lavage and/or granulocyte-macrophage colony-stimulating factor inhalation during the follow-up periods.

| Initial DSS | Final DSS | |  |
| --- | --- | --- | --- |
|  | DSS=1 | DSS≥2 |  |
|  | n | n | % |
| 1 | 7 | 1 | 87.5 |
| 2 | 6 | 6 | 50.0 |
| 3 | 0 | 2 | 0.0 |
| 4 | 0 | 1 | 0.0 |
| 5 | 0 | 0 | 0.0 |
| Total | 13 | 10 | 56.5 |

Abbreviations: DSS, disease severity score
