# Supplementary material for: A long-term observational study on autoimmune pulmonary alveolar proteinosis revealed a sustained and generalized decrease in serum autoantibody levels
Source: Orphanet J Rare Dis. 2026 Mar 11;21:190. doi: 10.1186/s13023-026-04274-w (PMC13159374; doi:10.1186/s13023-026-04274-w)
Supplement: Supplementary file 2 — Supplementary Material 2: Title: Binary logistic regression analysis of improved DSS for all patients Description: As described in the text. [file 13023_2026_4274_MOESM2_ESM.docx]

| **Table S2** Binary logistic regression analysis of improved DSS for all patients | | | | | | | |
| --- | --- | --- | --- | --- | --- | --- | --- |
|  |  | Univariate | | | Multivariate | | |
|  | reference | odds ratio | 95% CI | p value | odds ratio | 95% CI | p value |
| Gender | man | 1.400 | 0.518-3.782 | 0.507 |  |  |  |
| Age at onset |  | 0.998 | 0.966-1.031 | 0.896 |  |  |  |
| Smoking status | non-smoker | 0.804 | 0.394-1.640 | 0.549 |  |  |  |
| Initial DSS | DSS 1 | 4.696 | 1.927-11.446 | 0.001 | 12.126 | 3.198-45.987 | <0.001 |
| Comorbid autoimmune disease | none | 0.894 | 0.183-4.364 | 0.890 |  |  |  |
| Fibrotic patterns on CT | none | 0.353 | 0.108-1.150 | 0.084 | 0.032 | 0.002-0.563 | 0.019 |
| %FVC |  | 1.030 | 1.001-1.061 | 0.045 | 1.071 | 1.020-1.125 | 0.006 |
| FEV1/FVC at diagnosis |  | 0.978 | 0.913-1.049 | 0.538 |  |  |  |
| %DLCO at diagnosis |  | 0.990 | 0.964-1.016 | 0.443 |  |  |  |
| Serum KL-6 levels at diagnosis | 100 U/ml incease | 1.001 | 0.995-1.007 | 0.827 |  |  |  |
| Serum KL-6 levels at final visit | 100 U/ml incease | 0.987 | 0.967-1.007 | 0.209 |  |  |  |
| Serum αGM levels at diagnosis |  | 0.998 | 0.994-1.002 | 0.347 |  |  |  |
| Serum αGM levels at final visit |  | 0.993 | 0.981-1.005 | 0.236 |  |  |  |
| GM-CSF inhalation | not done | 1.429 | 0.531-3.842 | 0.480 |  |  |  |
| Corticosteroids therapy | not done | 1.250 | 0.324-4.826 | 0.746 |  |  |  |
| Whole lung lavage | not done | 1.244 | 0.463-3.347 | 0.665 |  |  |  |
| Long-term oxygen therapy | not done | 1.316 | 0.456-3.799 | 0.612 |  |  |  |
| Follow-up periods from the onset |  | 1.000 | 0.898-1.114 | 0.999 |  |  |  |

Abbreviations: DSS, disease severity score; CT, computed tomography; CI, confidence interval; FVC, forced vital capacity; FEV1, forced expiratory volume in one second; DLCO_,_ diffusing capacity of the lung for carbon monoxide; KL-6, Krebs von den Lungen-6; αGM, anti-granulocyte-macrophage colony-stimulating factor IgG autoantibody; GM-CSF, granulocyte-macrophage colony-stimulating factor.
