# Supplementary material for: A long-term observational study on autoimmune pulmonary alveolar proteinosis revealed a sustained and generalized decrease in serum autoantibody levels
Source: Orphanet J Rare Dis. 2026 Mar 11;21:190. doi: 10.1186/s13023-026-04274-w (PMC13159374; doi:10.1186/s13023-026-04274-w)
Supplement: Supplementary file 3 — Supplementary Material 3: Title: Binary logistic regression analysis of death due to aPAP for all patients Description: As described in the text. [file 13023_2026_4274_MOESM3_ESM.docx]

| **Table S3** Binary logistic regression analysis of mortality due to aPAP for all patients | | | | |
| --- | --- | --- | --- | --- |
|  | Univariate | | | |
|  | reference | odds ratio | 95% CI | p value |
| Gender | man | 2.880 | 0.24-33.511 | 0.398 |
| Age at onset |  | 0.981 | 0.915-1.052 | 0.597 |
| Smoking status | non-smoker | 2.123 | 0.456-9.877 | 0.337 |
| Initial DSS | DSS 1 | 1.581 | 0.570-4.386 | 0.379 |
| Comorbid autoimmune disease | none | 4.583 | 0.36-58.352 | 0.241 |
| Fibrotic patterns on CT | none | 3.23E+08 | 0.000- | 0.997 |
| %FVC |  | 0.954 | 0.897-1.015 | 0.136 |
| FEV1/FVC |  | 0.979 | 0.839-1.142 | 0.784 |
| %DLCO |  | 0.943 | 0.871-1.020 | 0.145 |
| Serum KL-6 levels at diagnosis | 100 U/ml increase | 1.001 | 0.990-1.012 | 0.828 |
| Serum KL-6 levels at final visit | 100 U/ml increase | 1.041 | 1.012-1.070 | 0.006 |
| Serum αGM levels at diagnosis |  | 1.000 | 0.992-1.008 | 0.986 |
| Serum αGM levels at final visit |  | 0.996 | 0.949-1.045 | 0.870 |
| GM-CSF inhalation | not done | 1.79+E08 | 0.000- | 0.998 |
| Corticosteroids therapy | not done | 2.889 | 0.237-35.285 | 0.406 |
| Whole lung lavage | not done | 1.86+E08 | 0.000- | 0.998 |
| Long-term oxygen therapy | not done | 2.85+E08 | 0.000- | 0.997 |
| Follow-up periods from the onset |  | 0.846 | 0.582-1.231 | 0.382 |

Abbreviations: aPAP, autoimmune pulmonary alveolar proteinosis; DSS, disease severity score; CT, computed tomography; CI, confidence interval; FVC, forced vital capacity; FEV1, forced expiratory volume in one second; DLCO, diffusing capacity of the lung for carbon monoxide; GM-CSF, granulocyte-macrophage colony-stimulating factor; KL-6, Krebs von den Lungen-6; αGM, anti-granulocyte-macrophage colony-stimulating factor IgG autoantibody
