# Supplementary material for: A long-term observational study on autoimmune pulmonary alveolar proteinosis revealed a sustained and generalized decrease in serum autoantibody levels
Source: Orphanet J Rare Dis. 2026 Mar 11;21:190. doi: 10.1186/s13023-026-04274-w (PMC13159374; doi:10.1186/s13023-026-04274-w)
Supplement: Supplementary file 4 — Supplementary Material 4: Title: Distribution of DSS stratified by each treatment modality and assessment phase in patients with initial DSS>1. Description: As described in the text. [file 13023_2026_4274_MOESM4_ESM.docx]

| **T****able S4 Distribution of DSS stratified by each treatment modality and assessment phase in patients with initial DSS>1.**   1. **GM-CSF inhalation B. WLL** | | | | | | | | | | | | | | | | |
| --- | --- | --- | --- | --- | --- | --- | --- | --- | --- | --- | --- | --- | --- | --- | --- | --- |
|  |  | Initial DSS | | Maximum DSS | | Final DSS | |  |  |  | Initial DSS | | Maximum DSS | | Final DSS | |
|  |  | GM-CSF inhalation | | | | | |  |  |  | WLL | | | | | |
| DSS |  | none | done | none | done | none | done |  | DSS |  | none | done | none | done | none | done |
| 1 | n | - | - | 2 | 0 | 10 | 8 |  | 1 | n | - | - | 2 | 0 | 8 | 10 |
|  | % | - | - | 8.3% | 0.0% | 41.7% | 32.0% |  |  | % | - | - | 8.3% | 0.0% | 33.3% | 40.0% |
| 2 | n | 19 | 13 | 8 | 0 | 12 | 13 |  | 2 | n | 14 | 18 | 7 | 1 | 13 | 12 |
|  | % | 79.2% | 52.0% | 33.3% | 0.0% | 50.0% | 52.0% |  |  | % | 58.3% | 72.0% | 29.2% | 4.0% | 54.2% | 48.0% |
| 3 | n | 4 | 6 | 6 | 4 | 0 | 2 |  | 3 | n | 7 | 3 | 6 | 4 | 1 | 1 |
|  | % | 16.7% | 24.0% | 25.0% | 16.0% | 0.0% | 8.0% |  |  | % | 29.2% | 12.0% | 25.0% | 16.0% | 4.2% | 4.0% |
| 4 | n | 1 | 4 | 5 | 13 | 2 | 0 |  | 4 | n | 2 | 3 | 6 | 12 | 2 | 0 |
|  | % | 4.2% | 16.0% | 20.8% | 52.0% | 8.3% | 0.0% |  |  | % | 8.3% | 12.0% | 25.0% | 48.0% | 8.3% | 0.0% |
| 5 | n | 0 | 2 | 3 | 8 | 0 | 2 |  | 5 | n | 1 | 1 | 3 | 8 | 0 | 2 |
|  | % | 0.0% | 8.0% | 12.5% | 32.0% | 0.0% | 8.0% |  |  | % | 4.2% | 4.0% | 12.5% | 32.0% | 0.0% | 8.0% |
|  | n | 24 | 25 | 24 | 25 | 24 | 25 |  |  | n | 24 | 25 | 24 | 25 | 24 | 25 |
| p value^a^ | | 0.16 | | <0.001 | | 0.23 | |  | p value^a^ | | 0.48 | | <0.001 | | 0.51 | |
|  |  |  |  |  |  |  |  |  |  |  |  |  |  |  |  |  |
| Abbreviations: DSS, disease severity score; WLL, whole lung lavage. ^a^ Fisher's exact test. | | | | | | | | | | | | | | | | |
